# The Role of Social Support in Adolescent/Young Adults Coping with Cancer Treatment

**DOI:** 10.3390/children7010002

**Published:** 2019-12-23

**Authors:** Sarah Pennant, Simon C. Lee, Suzanne Holm, Kelli N. Triplett, Laura Howe-Martin, Ryan Campbell, Julie Germann

**Affiliations:** 1 Children’s Medical Center, Dallas, TX 75235, USA; suzanne.holm@childrens.com (S.H.); kelli.triplett@childrens.com (K.N.T.); ryan.campbell@childrens.com (R.C.); 2 UT Southwestern Medical Center, Dallas, TX 75390, USA; simoncraddock.lee@UTSouthwestern.edu (S.C.L.); laura.howe-martin@UTSouthwestern.edu (L.H.-M.); 3 UT Southwestern Simmons Comprehensive Cancer Center, Dallas, TX 75390, USA; 4 UT Southwestern Moncrief Cancer Institute, Fort Worth, TX 76104, USA

**Keywords:** Adolescent/Young Adult (AYA), cancer, social support, qualitative, on-treatment

## Abstract

Adolescents/young-adult (AYA) cancer patients are a psychosocially at-risk group as they are often less well-studied than other age cancer cohorts. Therefore, they experience disparities in access to developmentally informed treatment. Social support has been determined as an important aspect of AYAs’ cancer experience, but additional research was needed to describe specific behaviors AYAs found helpful and to explore how AYAs seek opportunities for additional support. As part of a larger qualitative study, study aims were to determine how AYAs (ages 15–26) cope during cancer treatment and examine how social support interacts with individual AYA coping. Participants included 10 AYA cancer patients undergoing treatment (mean age = 18.9 years) and 10 parents (mean age = 45.6 years). Descriptively, participants scored within the normal to high range on measures of hope, depression/anxiety/stress, quality of life, and social support. Participants completed semi-structured, audio-recorded interviews that were transcribed and coded as generated. Qualitative analysis was guided by principles of grounded theory and utilized the constant comparative approach. Themes within social support groups included presence, distraction, positive attitude, and maintaining AYA autonomy, communication, and advocacy. Results suggest social supports provide additional coping resources for AYAs with cancer through supplementing individual coping strategies. Future directions/implications for intervention/treatment are discussed.

## 1. Introduction

While the number of medical advancements and research studies have led to increases in pediatric and adult cancer survival rates throughout the past four decades, the survival rates for adolescent young adult (AYA; ages 15–39) cancer patients have not demonstrated the same levels of improvement [1]. This cancer population has been less studied and understood in comparison to other age cohorts [1] and, therefore, lack access to care tailored to their unique developmental needs. While scientific advancement to improve medical outcomes and survival rates for this cohort is warranted, research addressing AYAs’ psychological, social, and emotional preferences during cancer treatment is equally important to improve AYAs’ quality of life and overall functioning.

Little is known about AYA cancer patients’ on-treatment experience and AYA survivors’ long-term psychological outcomes. AYAs appear to be an at-risk cohort of cancer patients, as adult survivors diagnosed during adolescence report significantly more mood symptoms [2], poorer health-related quality of life [3,4], lower scores of health competence (e.g., health perceptions, cognitive competence, and autonomy), significantly greater psychological distress, fewer positive health beliefs [5], and more social problems [6] than individuals diagnosed during school age (ages 6–12) or earlier. Even for medically healthy and typically developing individuals, the adolescent developmental stage poses distinct challenges, which can be further complicated by the introduction of a chronic illness such as cancer [6,7,8].

Social support through family, friends, healthcare providers, and other individuals [8] appears to be one of the most important and beneficial aspects for adolescents and young adults undergoing cancer treatment. Both parental and friend/peer support appear to have a unique and important influence on AYAs coping with cancer, but findings are mixed about which of these relationships are most meaningful [9,10,11,12,13]. Social support has been shown to be important for most cancer populations; however, few studies have explicitly examined which actions and behaviors are the most helpful, particularly for AYAs undergoing treatment. Furthermore, fewer studies describe behaviors from a wide variety of social supports, and even fewer examine findings communicated directly from AYAs themselves.

As part of a larger qualitative project focused on coping and hope theory in AYA cancer patients, researchers examined how a wide range of social supports provide assistance for this developmentally at-risk population. This study gathered data directly from AYA cancer patients using semi-structured interviews to explore specific actions helpful to AYA patients, what behaviors they would like from their social supports, and what advice they would give to others in their situation. The purposes of these findings were to inform the development of future psychosocial interventions tailored for AYAs; to provide guidance for family, friends, cancer peers, medical teams, and other AYA patients; and to empower AYA patients to communicate their individual needs to social supports.

## 2. Materials and Methods

### 2.1. Setting

This study was conducted at Children’s Health, a pediatric hospital within an academic medical center in Dallas, Texas, which offers children, adolescents, and young adults multidisciplinary psychosocial support during cancer treatment. This qualitative report was part of a larger qualitative study examining the role of hope and social support in adolescents coping with cancer treatment.

### 2.2. Participants

**Figure 1:**
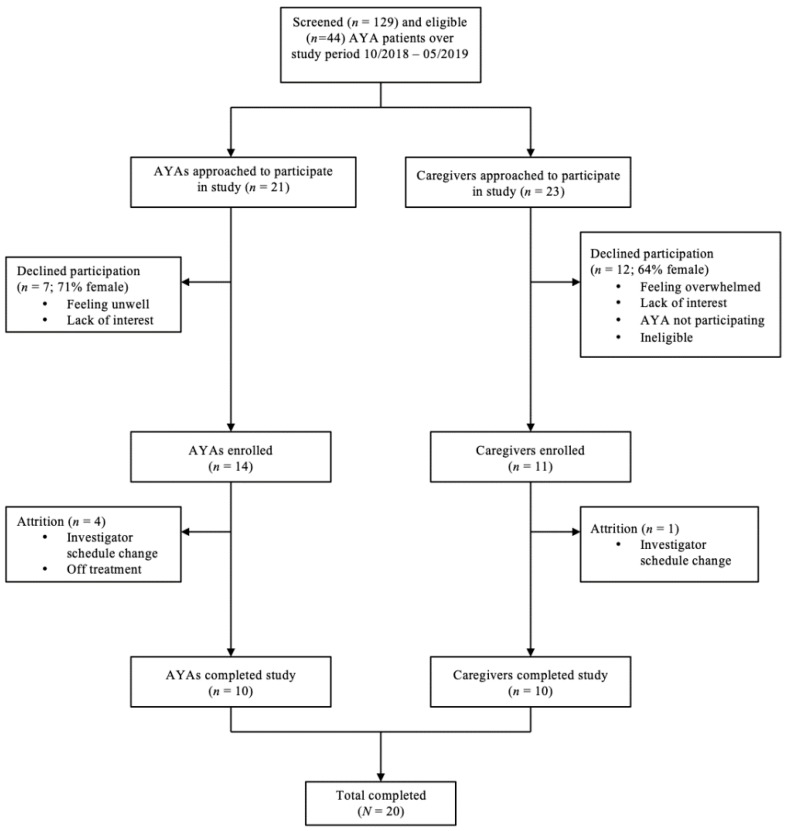
Consort diagram of participant flow.

Inclusion criteria for the AYA group was as follows: (1) English-speaking, (2) age of 15–29 years, and (3) currently receiving medical treatment (at any time point) at the cancer center for any cancer diagnosis. Inclusion criteria for the caregiver group was as follows: (1) English-speaking, (2) primary caregiver for an AYA patient, and (3) AYA child/spouse is receiving medical treatment (at any time point) at the cancer center for any cancer diagnosis. Exclusion criteria included (1) patients and/or caregivers displaying symptoms of delirium or psychosis that precluded their ability to participate in the qualitative interview or provide consent/assent, and (2) patients and/or caregivers with intellectual disability that precluded their ability to participate in the qualitative interview or provide consent/assent. Caregivers could participate even if their AYA child did not. “Treatment” was defined as active chemotherapy and/or radiation, or less than 100 days post-bone marrow transplant. Treatment status was determined through review of patient electronic medical records (EMRs). Over the course of 7 months, 14 AYAs and 11 parents were enrolled (see Figure 1).

A total of 10 AYA patients [mean (*M*) age = 18.9 years] and 10 parents (*M* age = 45.6 years) completed the study. Participant demographic information (e.g., age, sex, race, ethnicity, insurance status, marital status, and education level) is described in Table 1. AYA participant diagnoses included Acute Lymphocytic Leukemia (ALL; 40%), Hodgkin’s Lymphoma (20%), Medulloblastoma (10%), Ewing’s Sarcoma (10%), Nerve Sheath Tumor (10%), and Osteosarcoma (10%). Two AYA participants were being treated for cancer relapse and one was being treated for secondary cancer. Type of treatment included chemotherapy only (60%), a combination of chemotherapy and radiation (30%), and chemotherapy/radiation/surgery (10%). At the time of interview, median time since diagnosis (or relapse) was 71.5 days [*M* = 158.8 days; standard deviation (*SD*) = 203.8 days; range 18–713 days] for the AYA group. Four AYA-parent dyads participated in the study, thus the AYAs of the parent participants were not all the same group as the AYA participants. One AYA participant was married and one was engaged. Accordingly, parents’ AYA children were diagnosed with ALL (40%), Hodgkin’s Lymphoma (20%), Osteosarcoma (20%), Nerve Sheath Tumor (10%), and Chronic Myeloid Leukemia (CML; 10%). Two AYA children of parent participants were being treated for cancer relapse (*n* = 1) or secondary cancer (*n* = 1). Type of treatment for AYAs of the participants included chemotherapy only (70%), a combination of chemotherapy and radiation (20%), and bone marrow transplant (10%). At the time of interview, median time since diagnosis (or relapse) for the AYAs of the parent participants was 161 days (*M* = 369.7 days; *SD* = 402 days; range 28–1196 days) for the caregiver group.

Hollingshead four-factor scores [14] for family socioeconomic status (SES) were created with occupational status and highest level of education attained by parents or AYAs living in the household during recruitment. Scores for family SES ranged from low- to high-class (12–53.5), while mean score for family SES was 37 (*SD* = 12.8), indicating on average a middle-class family SES. The participants that were AYAs did not differ by demographic variables (e.g., age, sex, marital status, education status, insurance status) when compared to the AYAs of the caregiver group. The participants that were AYAs also did not differ by demographic variables (e.g., age, sex, education status, insurance status) when compared to the caregiver group.

### 2.3. Procedure

All study procedures were approved by the UT Southwestern Institutional Review Board (STU082018-023) with additional site approval by Children’s Health Office of Research Administration. Eligible patients and/or caregivers were approached in private outpatient, day hospital, or inpatient rooms. Informed consent was obtained from all participants prior to data collection. Participants were not compensated for their participation. Participation involved two components: (1) completion of paper-and-pencil questionnaires (at initial visit and directly following the interview) and (2) completion of an approximate 1–2 h semi-structured interview during a subsequent clinic visit or hospital stay. All interviews were audio-recorded and transcribed verbatim.

Participants’ treatment consisted of a combination of inpatient and outpatient care; accordingly, interviews with AYAs were completed in one of three primary locations: (1) inpatient hospital rooms (*n* = 6), (2) outpatient day hospital rooms (*n* = 3), or (3) outpatient clinic room (*n* = 1). Interviews with caregivers were conducted in one of the following locations: (1) available private area during child’s inpatient hospitalization (*n* = 5), or (2) waiting area or private consultation room in day hospital floor (*n* = 5). Interviews took place during the same day that consent was obtained (*n* = 6) and up to 27 days afterwards (median = 0 days, mode = 0 days, *M* = 6.5 days).

### 2.4. Measures

Demographic information. The socio-demographic questionnaires were researcher-created and included questions regarding preferred child language/preferred parent language, parent/child education level, parent/child employment status, household makeup, patient/child relationship status, and perception of patient/child friendships. Researchers recorded the following demographic information from patient electronic medical record review: age, sex, date of birth, diagnosis, date of diagnosis, race, ethnicity, and insurance status (private versus public). Each participant completed a demographic questionnaire.

Hope. The Adult Hope Scale, titled “The Goals Scale” for participants, (AHS) [15] is a 12-item self-report measure for individuals 15 years and older. Four questions address agency (e.g., “I meet the goals that I set for myself”), four items address pathways (e.g., “I can think of many ways to get the things in life that are most important to me”), and four items are filler items (e.g., “I feel tired most of the time”). The scale generates three scores: an overall hope score (range score of 8–64) combining agency and pathways items, or individual agency and pathways subscores (range score of 4–32). Snyder et al. [16] reported good levels of internal reliability (alphas of 0.74–0.84 for overall scale, and 0.63–0.80 and for the agency and pathways factors) and factor structure. Concurrent, discriminant, and convergent validity has been supported in research [15]. The Adult Hope Scale test-retest correlations were 0.85 (*p* < 0.001), over a 3-week interval, 0.73 (*p* < 0.001) over an 8-week interval, and 0.76–0.82 (*p* < 0.001) in a 10-week sample. All participants completed the AHS assessing their individual hope level.

Depression and anxiety. The Depression Anxiety Stress Scale-21 (DASS-21) [17] is a 21-item self-report screening tool for identifying, differentiating, and assessing depression (e.g., “I couldn’t seem to experience any positive feeling at all”), anxiety (e.g., “I felt scared without any good reason”), and stress (e.g., “I tended to over-react to situations”). Each item is scored on a four-point scale (0 = “does not apply to me at all”, 3 = “applies to me very much or most of the time”) and the sum of all items generates a total score, while the sum of seven items generates the subscale score. Higher scores indicate greater levels of distress. Separate cut-off scores and corresponding descriptive categories (e.g., “normal” to “extremely severe”) are provided for depression, anxiety, and stress subscales [17]. Good internal consistencies have been supported in research (alphas of 0.79 to 0.97 for subscales), and the three subscales have been shown to have positive relationships with one another [18,19]. The DASS-21 depression and anxiety subscales have been shown to have good convergent validity [18]. Construct validity for the depression and anxiety subscales have been determined in adolescent samples (ages 11–15), while the stress subscale needs further examination in this age group [19]. Each AYA participant completed the DASS-21.

Quality of life. The Pediatric Quality of Life Inventory 3.0—Cancer Module (PedsQL-C) [20] is a 27-item self-report and parent-proxy instrument that measure health-related quality of life (HRQOL) in teens (ages 13–18), young adults (ages 18–25), and adults (older than 25) with cancer. Parents report their perception of their child’s HRQOL; items are essentially identical to child self-report prompts. The PedsQL-C queries how much of a problem each item has been during the past month. Participants and parents are instructed to answer with a five-point response scale (0 = never a problem; 1 = almost never a problem; 2 = sometimes a problem; 3 = often a problem; 4 = almost always a problem). Items are reverse-scored and transformed to a scale 0–100 (0 = 100; 1 = 75; 2 = 50; 3 = 25; 4 = 0) so that higher scores indicate better HRQOL. The measure encompasses the following eight subscales: pain and hurt (e.g., “I hurt a lot”), nausea (e.g., “I feel too sick to my stomach to eat”), procedural anxiety (e.g., “I get scared when I have to have blood tests”), treatment anxiety (e.g., “I get scared when I am waiting to see the doctor”), worry (e.g., “I worry about the side effects from medical treatments”), cognitive problems (e.g., “I have trouble writing work/school papers or reports”), perceived physical appearances (e.g., “I feel I am not good looking”), and communication (e.g., “It is hard for me to ask the doctors and nurses questions”). Studies support good internal reliability (alphas of 0.70–0.93 for individual scale self-report and alpha of 0.79–0.98 for scale parent-proxy) [20,21]. Construct validity has been determined in children (ages 5–18) and young adults (ages 16–25) with cancer [20,21]. Each participant completed the PedsQL-C; however, AYA participants completed the inventory describing their self-reported HRQOL, while caregiver participants completed the inventory about their AYA child’s HRQOL.

Social support. Multidimensional Scale of Perceived Social Support (MSPSS) [22] is a 12-item self-report scale validated for ages 15–42 that measures the perceived adequacy of support from family (e.g., “My family really tries to help me”), friends (e.g., “I can count on my friends when things go wrong”), and significant others (e.g., “There is a special person in my life who cares about my feelings”). It uses a seven-point Likert-type format (1 = very strongly disagree; 7 = very strongly agree). The authors report good internal reliability (0.81–0.90 for family subscale, 0.90–0.94 for friends subscale, 0.83–0.98 for significant other subscale, and from 0.84 to 0.92 for the scale as a whole) [22]. Test-retest correlations ranged from 0.75 to 0.85. Each AYA participant completed the MSPSS.

Semi-structured interview (Appendix A). Each participant completed an audio-recorded, semi-structured interview. Interview topics for patients included patient’s overall coping/experience of cancer treatment (e.g., “Tell me how things have gone for you since you were diagnosed with cancer.”) with follow-up questions related to individual coping (e.g., “What kind of things are you doing/have you done to get through this time?”) and social support (e.g., “What have others done? Why was it unhelpful/helpful? What do you wish they would/would not do?”). The second interview topic included patient’s beliefs about hope (e.g., “However you think of hope, how is hope related to all of this?”). Interview topics for parents/caregivers included caregiver perceptions of their child’s overall coping/experience of cancer treatment (e.g., “Tell me how things have gone for your child since their diagnosis.”), caregiver perceptions of their child’s social support during treatment (e.g., “What have you done to help your child through this time?” and “What have others done?”), and parent/caregiver’s belief about hope and it’s relation to coping (e.g., “However you think of hope, how is hope related to all of this?”). Throughout study process, questions were added to interview guide as themes emerged from interviews. The interview process allowed researchers to discuss emerging themes, follow up on topics discussed by interviewees, and confirm/clarify themes described by earlier interviewers (e.g., member-checking).

### 2.5. Data Analysis

Consistent with the aims of our qualitative study, responses to the battery of measures were used to describe overall sample makeup and not quantitatively analyzed. Transcripts were initially reviewed without coding in order to identify emerging themes [23]. All transcripts were uploaded and coded in a qualitative data analysis computer software, NVivo 12 Mac (QSR International, AUS), to ensure consistent and reliable code application. Guided by the principles of grounded theory methodology [24], iterative processes were used during data analysis. The investigator openly coded each interview to define key concepts and developed a preliminary code structure after the first two interviews. Corbin and Strauss [25] defined open coding as the process of breaking down, examining, comparing, conceptualizing, and categorizing data. Using the constant comparative approach, investigators applied this code structure to subsequent transcripts and revised codes and themes as new concepts emerge [23,24,26]. Therefore, transcripts were reviewed and coded prior to conducting subsequent interviews so that analysis continually informed data collection [23,24,26]. Investigators recorded their own internal reactions and processes via memoing throughout the data collection and analysis process. Investigators continued this process of interviewing and analysis until thematic saturation was reached (e.g., no new ideas emerged from subsequent interviews) [24,26]. Investigators resolved coding uncertainties through discussion to ensure agreement in coding structure. Investigators discussed and interpreted themes from data with the guidance of a qualitative expert and clinician support.

Credibility was determined by debriefing and sharing summary findings with study participants during interview process (e.g., “member-checking” [27]) to allow for clarification and addressing inconsistencies. Transferability and transparency were determined by including detailed descriptions of the setting, context, and participants. Dependability and confirmability were determined by auditing from investigators, a qualitative methods expert, and a clinician with expertise in working with pediatric/AYA cancer populations.

## 3. Results

### 3.1. Results from Instruments/Measures

Results from the measures of hope, depression/anxiety/stress, HRQOL, and social support are described in Table 2. The scores described in this section refer to the scores obtained prior to the interview at the time of study enrollment, as scores did not change between pre- and post-interview. On the AHS, both AYA and parent groups reported overall high levels of total hope. On the DASS-21, AYAs’ mean reported depressive and stress symptoms scores fell within the normal range, while mean anxiety scores fell in the mild range. For cancer-related quality of life (PedsQL-C), AYAs generally reported higher health related quality of life than parent-report of AYA HRQOL, although all scores fell within the average range. On the MSPSS, AYAs reported high levels of total social support, including high levels of family, friend, and significant other support.

### 3.2. Results from Qualitative Analysis

Here, we focus on the responses describing social support, although results of the larger study examined AYA coping and hope in addition to social support. Participants consistently highlighted the importance of social support, describing it as the most helpful aspect during their cancer experience. One participant expressed this view succinctly when he said; “You can’t get through it alone. That is, I mean, that is impossible.” Furthermore, no participants responded to the prompts about coping (Appendix A) without describing social support. Participants described sources of social support, which included family, friends, cancer peers, and other sources (e.g., medical team, colleagues, teachers, organizations, social media, communities, and athletic teams). All themes and categories are listed in Table 3, Table 4, Table 5, Table 6 with quotes providing examples of each category and the participant identifier.

We present findings by source of social support and the described specific actions/behaviors of these sources, while recognizing that similar themes emerged within multiple sources (e.g., presence). Responses are divided into tables based on source of support, as three primary categories of social support emerged: family (see Table 3), friends (see Table 4), and others (see Table 5). Our findings also describe others’ actions and behaviors that participants felt were not helpful during treatment, as well as participants’ advice and recommendations for other AYAs and/or social supports (see Table 6). For all tables, quotes utilize the corresponding participant identifiers: “A” = AYA participant, “C” = Caregiver participant, and numbers represent the unique participant codes.

#### 3.2.1. Family

One of the primary sources of social support that AYAs described was from family members (e.g., mothers, fathers, siblings, spouses, and extended family members; see Table 3). Most participants commented on how important family support was for their coping process. Although various family members were described when discussing familial support, mothers were most often noted as the most important source of social support. Supportive actions from fathers, siblings, cousins, grandparents, and aunts were also reported and are included in the findings. Five themes emerged regarding helpful family social support: providing physical and emotional presence, advocating for AYA’s needs/providing additional information related to treatment, maintaining AYA autonomy (when appropriate), modeling a positive attitude, and assisting in everyday tasks.

##### 3.2.1.1. Presence

Every participant relayed the benefit and value of having a family member present throughout treatment. Although primarily described as physical presence or company during hospitalizations or at home, others also described the value in having family members emotionally available in the event they wish to process events with someone. Although this was primarily reported when discussing social support from family members, participants also relayed the importance of other sources of social support being present as well, such as friends, medical staff, peers, and members of the community. The findings describing these sources of support will be discussed in the sections below.

##### 3.2.1.2. Everyday Tasks

When family members and caregivers are physically present, AYAs relay the benefit of having assistance with everyday tasks at home and in the hospital. These supportive behaviors appear to alleviate challenges due to treatment side effects (e.g., pain, fatigue, and nausea). Many participants also described the importance of having family members bring them food in the hospital or at home.

##### 3.2.1.3. Maintaining Autonomy (Independence, Privacy, and Space)

Although physical and emotional presence was reported as important behaviors from social supports, participants conversely relayed the value in maintaining autonomy (e.g., fostering independence and allowing privacy and space). Understandably, these categories that fell under autonomy were also aspects that AYAs described as developmentally related challenges that are interrupted by a cancer diagnosis. Participants described how family members address these challenges by providing space and privacy appropriately during treatment in order for the AYA patient to maintain some sort of independence, in an otherwise very dependent situation. Some parents even described ways they planned to increase independence and autonomy for their AYA child during treatment.

##### 3.2.1.4. Advocate/Source of Information

When describing how family members were helpful and what specific actions were useful, many AYAs relayed the importance of having a parent advocate for their needs to other individuals, including medical team staff, other family members, or friends. Although AYA’s self-driven advocacy was described as a useful individual coping strategy, participants described how parents had a more active role in advocating and communicating the AYA patient’s needs, being more aware of questions or information that needed to be addressed, and vocalizing these needs to whomever appropriate. AYAs and parents also described the importance of having a parent record information and clarify treatment questions, particularly when the AYA patient was feeling unwell or fatigued.

##### 3.2.1.5. Positive Attitude

Modeling/maintaining a positive attitude and using comic relief throughout treatment were described as helpful family behaviors, despite how the AYA is feeling or what they are thinking. For example, one AYA described the importance of continuing to make jokes with her parent, even if melancholic, allowed her to stay positive and positively impacted her overall mood. AYAs also described the positive impact that a caregiver’s positive attitude has on their individual coping.

#### 3.2.2. Friends

Friends were another source of social support that AYAs and parents described as an important part of coping with treatment (see Table 4). Some patients also described the disappointment with friends disappearing after their diagnosis or reigniting friendships with individuals from the past, highlighting the importance of consistent friend support during treatment. Some of these acts of support are similar to those described as helpful family behaviors, while others appear distinct for friends of AYAs. These categories include the importance of physical presence, maintaining communication and contact, providing a source of distraction or normalization, and sending words of encouragement. Suggestions for how AYAs felt their friends could be more helpful will be addressed in sections below.

##### 3.2.2.1. Presence

As discussed in the family support, (Section 3.2.1.1) the physical and emotional presence from friends is also important for AYAs coping with cancer treatment. Participants relayed that friends’ physical presence was helpful as it allowed the AYA patients to feel less alone during their treatment process and helped maintain a sense of normalcy, both of which were also described as challenges for AYAs after cancer diagnosis. Parents also noted that interaction with friends appeared to positively impact their AYA child’s mood.

##### 3.2.2.2. Communication/Contact

Participants also reported that staying in touch and maintaining communication was helpful when friends were not able to be physically present. AYAs reported this was helpful in order to stay updated on their friends’ lives and to feel included, despite treatment limitations. They reported this contact was typically maintained via phone (e.g., texting, calling, video chatting) and was helpful in sustaining the sense of support, even when patients were in the hospital or home alone.

##### 3.2.2.3. Distraction

Participants described value in interacting and participating in activities with friends, as it provided opportunities for positive distraction. These activities and interactions were described as helpful, as they provided a sense of normalcy for the AYAs patients during treatment. Many activities were described and included watching movies, playing video games, bringing/eating meals, and talking.

##### 3.2.2.4. Encouragement/Gestures

Participants also described how friends’ encouraging words and gestures were meaningful during their cancer diagnosis. Encouraging behaviors included sending letters, giving posters from school, surprising the AYA patient with favorite games or food, and sending supportive messages (via phone or in person). Two AYAs also relayed that although they felt their friends could not truly understand their cancer experience, they appreciated their friends’ efforts to try to understand (e.g., asking questions to assist with deepening their knowledge of the AYA’s diagnosis).

#### 3.2.3. Other Social Support

Participants also identified and described actions from additional sources of social support other than family and friends, which were helpful during their cancer treatment. These included actions and support from (1) cancer peers, (2) medical staff, (3) spouses/significant others, (4) school or work, (5) community members, (6) cancer-specific organizations, and (7) professional or collegiate athletes (see Table 5).

##### 3.2.3.1. Peers

Peers, meaning other same-age cancer patients or survivors, were described as a unique and highly beneficial source of social support for AYA patients who had met them. Participants highlighted the value of having cancer peers as a resource for advice as well as a deeper level of understanding. Similarly, parents expressed that it was useful for them to consult with parents from school or the community who had a child go through cancer treatment, to help prepare them for the various challenges that typically arise.

For AYA patients who had not had the opportunity to meet same-aged peers, participants relayed the desire to meet them for similarly stated reasons. Most expressed feeling like this would be a very useful and valuable experience for them. Participants also described the desire to meet other AYAs going through a shared experience, as they often felt isolated and much older than other patients at the pediatric setting.

##### 3.2.3.2. Medical Staff

Participants frequently described how valuable members of their medical team had been throughout their treatment process. Participants described helpful actions from various medical staff including doctors, nurses, social workers, psychologists, child life specialists, therapy dogs, music/physical therapists, and volunteers. Behaviors that were described as helpful included medical team’s overall preparedness during treatment, providing useful and appropriate recommendations for treatment (e.g., side effects), accurate information and education about treatment, honesty about treatment outcomes, modeling a positive attitude throughout treatment, listening to patient preferences, and addressing AYA patients as adults. AYA patients found these actions helpful as they helped address some of the uncertainties and difficulties with adjusting to their diagnosis.

##### 3.2.3.3. Spouse/Significant Other

Three participants described social support from spouses/significant others. This type of support appeared to be a distinct type of supportive relationship with unique characteristics. All of these AYAs expressed a preference of seeking support for personal difficulties related to cancer treatment from their significant other (e.g., boyfriend, fiancé, wife) as opposed to their parents. Reported reasons for this preference were their significant others were better equipped to handle the patients’ distressing emotions as compared to patients’ parent or family members. They endorsed difficulty with expressing sadness and worries or discussing their illness with family, as they often felt that this would result in their family member becoming distressed. One of these participants also described how it was helpful for her significant other to have his own resources of social support, as it decreased guilt from expressing her distress.

##### 3.2.3.4. School/Work

Participants also described receiving support from school classmates/teachers or work colleagues/bosses. Some described specific actions of individuals at school, such as teachers or coaches visiting the patients during hospital admissions or organizing group letters and words of encouragement. AYAs also reported receiving help from bosses/teachers who were flexible with their absences and treatment limitations (e.g., guaranteeing employment after treatment ends, flexible with assignments) and who continued to check in with the AYA’s health status throughout treatment. Participants also described helpful actions from institutions as a whole, such as schools organizing a fundraiser at school or creating campaigns to support the AYA.

##### 3.2.3.5. Professional or Collegiate Athletes

AYAs described receiving support from professional or collegiate athletes during cancer treatment. These actions included teams providing opportunities to attend various sporting events or athletes visiting patients in the hospital. Participants reported that these actions positively impacted the AYA patients’ mood and helped provide positive distraction from treatment. Others relayed that teams and athletes sent words of encouragement, which helped them feel less isolated and alone during the cancer process.

##### 3.2.3.6. Organizations

AYA patients and parents discussed how helpful various local and/or national organizations were throughout the cancer experience. Participants relayed how various organizations allowed them to meet other cancer patients/survivors/families, provided useful information and assistance about specific aspects cancer or unique diagnoses (e.g., provided backpacks with necessary items for unexpected hospital admissions), and connected patients with financial assistance or other resources. In this way, organizations facilitated connection for AYA patients and families to other sources of social support (e.g., cancer peers, professional athletes).

##### 3.2.3.7. Community

Lastly, participants discussed how members in their community (e.g., church, neighborhood, parent groups) were helpful during cancer treatment. Some of the described actions were described by other sources of social support (e.g., providing financial assistance, fundraising, connecting to resources, sending words of encouragement, providing meals for the family). Other actions included providing support by helping with childcare for patient siblings or creating groups on social media to disseminate information about the AYA patient.

#### 3.2.4. Opinions/Advice

During the interview, participants were prompted with other questions about social support that weren’t necessarily related to specific sources of social support. Their responses still describe useful insight about the AYA cancer experience, but do not apply to the tables above as they discuss what actions or behaviors were not helpful during treatment and provide general advice to others (e.g., patients, families, medical team) about how to provide support (see Table 6).

##### 3.2.4.1. Not Helpful

Although many actions, activities, and interactions that AYAs and parents described were deemed as helpful or useful, some participants described actions or experiences that were not helpful during their cancer treatment from parents, friends, medical staff, and organizations. One of the most frequently described unhelpful behaviors was when others treated them differently after their cancer diagnosis, leading them to feel as though cancer was their only identity or that others pitied them. For example, some AYAs described wanting their friends and family to continue to joke with them, even tease them like before diagnosis, regardless if the jokes appear harsh. Joking or teasing may not be every patient’s baseline interpersonal dynamic, but most participants endorsed wanting to maintain the same type of relationship or communication style that existed prior to diagnosis.

Other actions that participants described as being unhelpful during treatment were when others expressed sadness or negative emotions around them regarding the AYA cancer diagnosis. Some AYAs relayed discomfort in seeing their parents become tearful or worried during their treatment, preventing them from wanting to share any of their experience in order to protect their family’s emotional functioning. Additional family factors, such as family discord related or unrelated to the AYAs cancer treatment, were described.

Additional described unhelpful actions or behaviors included searching about diagnoses/treatment on the Internet. Participants reported that when they searched for information about their diagnosis, they felt discouraged by articles and statistics, which may or may not have applied to their unique cancer situation. AYAs relayed that medical staff were helpful in addressing these concerns by directed them to trustworthy websites and articles and providing accurate, tailored information about their disease course. A few participants expressed disliking increased attention to the AYA during treatment, as this was something he or she had been uncomfortable with prior to diagnosis or because they did not want to ostracized for being “different” from other AYAs. Despite this discomfort, these AYAs still described helpful actions and behaviors of personal social supports (e.g., friends, family, medical staff) that were directed at the AYA patient versus support from organizations or larger community groups.

##### 3.2.4.2. Advice to Others

Participants also provided recommendations and suggestions for others based on what had been a challenge so far or how others could provide additional help. As described above, many described the desire to meet other same-age cancer patients or AYA cancer survivors, while others expressed interest in meeting younger cancer patients and to adopt a mentorship role. However, AYAs’ advice included increasing access to cancer peer support. Other recommendations were for additional sources of social support, such as AYA-specific resources, activities, or organizations, and tailoring resources to be more inclusive of adolescents and young adults in their helpful services. AYAs also expressed advice to their friends, requesting enhanced support, such as being treated the same as before treatment, increasing physical presence and emotional support, or asking questions so friends could gather a better understanding about the AYA’s cancer experience (Table 6).

## 4. Discussion

### 4.1. Social Support and AYA Coping

Although AYAs have demonstrated using an array of effective individual and self-coping mechanisms, social support and social coping appears to be the most helpful and important aspect for AYAs undergoing cancer treatment. The findings add to the existing literature about the importance of social support for AYAs undergoing cancer treatment [9,28,29,30] and provide guidance for clinical care when working with this age cohort. These findings also highlight how the suggestions for various sources of social support may differ in what behavior is helpful for AYA patients. The findings underscore the importance of strong social support from parents and friends, but also highlight how medical staff, organizations, and cancer peers can impact and improve psychosocial functioning during treatment.

#### 4.1.2. Family Support

The current study confirms that AYAs value the role of parental social support in their coping. The findings suggest parents of AYA patients must oscillate between being involved in and catering to their AYA child’s needs during treatment, while allowing space for perceived independence and autonomy. The cancer diagnosis and treatment itself may force AYAs to continue to depend on parents as physical and emotional demands can prove to be limiting. Although AYAs desire independence, they also value the involvement of parents for caregiving tasks, company, and advocacy during treatment. One study suggested that delays in adolescent independence were actually an adaptive strategy for adolescents with cancer, as it allowed AYAs to employ parents as the primary source of social support [31]. Perhaps AYAs lack the internal resources to cope during times of fatigue or nausea, so they regress to a reliance on parents as a way of getting through treatment.

AYA patients also value when parents model a positive attitude. Perhaps this parent behavior helps AYAs shift their focus to more positive cognitions and allows them to avoid their own worries or negative thoughts regarding their diagnosis and treatment. In fact, research has found that parental reassurance and distraction responses are associated with higher levels of posttraumatic growth after youth cancer treatment [32], highlighting parents’ role in how the AYA copes.

Although family support is an important part of AYAs coping with cancer, the current findings confirm that the relationship between AYA cancer patient and parent is unique, due to the delay in the separation process [33] and increased dependence on parents [5,31]. Research had found that enmeshment, rigidity, and overprotectiveness were maladaptive family dynamics during adolescent cancer treatment [34], and that adolescents struggled with feeling pressured to constantly communicate with parents [10]. AYAs in the current study relayed difficulty with seeing their family members become distressed during treatment, decreasing their likelihood to approach them for support. Perhaps by witnessing parental distress (e.g., sadness and anxiety), AYAs are forced to focus on the negative emotions and thoughts that they were previously actively avoiding. Additionally, if parents are struggling to cope themselves, then they might not provide the AYA with distracting activities and are unable to model positive attitudes. This interaction might explain the link between parent and child distress levels. One study found that pediatric cancer patients whose parents were distressed were more likely to be distressed themselves [35].

AYAs may choose to avoid sharing challenges with their parent to decrease the chance of their own emotional disturbance. Indeed, research has indicated that AYAs find it difficult to talk about their future or death with parents during treatment [11]. When parents express distress, they may be shifting the focus to AYA’s level of distress, something that is typically avoided for resilient AYA cancer patients. Due to parental emotional sensitivity, findings from the current study suggest that some AYAs prefer to rely on friends or significant others for emotional support.

#### 4.1.3. Friend Support

AYAs expressed the importance of support from friends. AYAs struggle with feeling as though their lives are at a standstill due to cancer treatment, particularly when comparing their lives to those of their friends and peers. Fostering peer relationships, experiencing romantic relationships, and making decisions about career/educational paths are a typical part of the AYA developmental trajectory [6,7,8], which explains why witnessing others undergo these experiences, and being distant from these experiences, are particularly challenging for AYAs with cancer. Maintaining connections and communication with friends throughout treatment is a way of continuing to develop deep friendships and experiencing the social milestones of AYA development. Therefore, friend support provides a unique opportunity to address this challenge, distinct from the impact of parental support. In fact, research has determined that close friendships are important for adolescent cancer patients, as they foster acceptance and provide opportunities for patients to share experiences with individuals other than their parents [9]. Similarly, another study found that youth with cancer who report higher levels of friend connectedness are more likely to demonstrate high resilience and growth during treatment [12]. The findings from this study are consistent with these findings and highlight the importance of close friends during AYA cancer treatment.

Study findings suggest that AYAs appreciate support through various forms of communication, such as texting, phone calls, video chatting, and letters. Friends also provide developmentally appropriate activities for AYA patients, which serve as effective ways to redirect AYA patients’ attention and reduce distressing stimuli. Social interaction in general, regardless of platform, serves either as a distraction or a mechanism for friends to process distressing thoughts with AYAs. Similar to parental support, friends must also find the balance between seeking opportunities to develop further understanding of the AYA’s cancer experience, while still treating the AYA patient as “normal” as possible. Friends appear to be unique sources of social support as they can provide additional AYA-approved routes to distraction and can be an avenue for processing and meaning-making when AYAs may not feel comfortable exploring these topics with their parents.

#### 4.1.4. Cancer Peer Support

Many AYA patients highlighted the value in or the desire to meet other same-aged cancer peers during treatment. Study findings regarding the importance of cancer peer support is consistent with research that found 100% of young adults on treatment and 96% of young adults off-treatment ranked the opportunity to meet other young adult patients with, or survivors of, cancer as one of the top five needs at diagnosis, during treatment, and after treatment, as compared to the 50–62% who ranked family and friend support as a top need [36]. These results indicate that AYA cancer peers or survivors are unique pathways of social support. These relationships allow AYA patients to have a shared experience, increase understanding, and foster deep and meaningful friendships on treatment during a developmental period where acceptance and peer relationships are important. Cancer peers may also provide suggestions and useful additional routes to coping with cancer-specific challenges that parents, friends, or AYA patients may not be aware of themselves. Cancer peer support groups themselves are mechanisms to processing and making meaning of the cancer experience. A variety of avenues to implement cancer peer support, such as in-person weekly meetings, weekend retreats, conferences, online forums, and therapeutic trips [37] have been explored in research. Despite the overwhelming interest in cancer peer support and differing ways to implement this type of support, starting and maintaining these programs still proves challenging [38]. Cancer peers appear to be a distinct group that could provide support through mutual understanding and provide a sense of normalcy during the AYA cancer experience.

#### 4.1.5. Medical Staff Support

Our participants indicated medical staff members have unique insight into the lives of AYA cancer patients and foster a sense of normalcy and acceptance for AYA cancer patients. Medical team members also have the ability to share valuable information to address AYA patients’ uncertainty about treatment and to assist with unpleasant side effects of treatment. They also provide encouragement and model positive attitudes for patients. Their insights into the AYA cancer experience allow them to normalize and validate these patients’ experience in a way that is different than other social supports. They also have the ability to disseminate information to all patients and be a resource during clinic visits or hospitalizations. Some participants described how medical team members provided effective distractions and advocated for patients to build connections with other social supports, like organizations, other families, or athletes. These findings demonstrate that medical staff members have the ability to intervene and improve coping through a number of pathways (e.g., distraction, encouragement, information).

#### 4.1.6. Other Social Support

Lastly, AYA cancer patients relayed helpful behaviors done by other social sources, such as cancer-specific organizations and camps, sports teams and professional athletes, and members of the community. These social supports impact mood and functioning through visiting patients in the hospital, providing novel experiences, and giving words of encouragement. The interactions and behaviors of these supports were often described as helpful and appreciated by AYA patients. These actions appear to serve as additional distractions or activities to which AYAs can refocus their attention (e.g., baseball game) versus ruminating on negative thoughts and emotions. Others helped provide novel activities that were approved by medical staff, which were unknown to patients and families beforehand. It appears that these forms of support provide additional avenues to coping and connecting with other cancer peers or groups. These other sources also provide words of encouragement or supportive actions, which participants describe as comforting to know others are thinking of them, that they are not forgotten or left behind at a time they may be feeling stuck.

### 4.2. Clinical Implications

Our study provides guidance for clinician intervention and considerations for families, friends, and medical staff when working with AYA cancer patients. The findings of our study detail the actions and behaviors that AYA patients have found helpful during their cancer experience. The results provide direction for clinical application, including increasing education/awareness about this patient cohort, focused screening, tailoring interventions, and adjusting administrative policies. We discuss these implications in the sections below.

#### 4.2.1. Implications for Education

The findings of our study provide information about the unique AYA cancer experience and carries implications for medical staff, as well as for AYA cancer patients themselves. First, we will discuss how these findings may increase awareness and inform the practice of medical providers. Most roles of medical team members are to address patient needs and treatment and provide information and/or suggestions when issues arise. Our study details information that allows medical staff to provide suggestions for psychosocial needs as they relate to emotional functioning. It also highlights the need for medical providers to be aware of various resources and recommendations to provide to AYA patients and families.

AYA cancer patients are treated in both adult and pediatric cancer centers. However, medical providers in both settings frequently lack experience in working with this age group and may not be attuned to the specific developmental challenges for these patients or how to best help them with their challenges [39]. Providers who work with children may be well versed in how parents of AYAs can provide support through presence and helping with daily tasks during cancer treatments, but may not consider how to respect patient autonomy/independence or provide guidance for AYAs’ significant others. Regardless of clinical experience, all providers can benefit from having additional information, education, and suggestions to distribute to AYA patients and families. As highlighted in this study, clinicians also ought to be aware of the local and national resources, organizations, sports teams, outreaches, and/or camps that serve AYA cancer patients in order to facilitate social support. Our study also provides suggestions for ways medical staff can improve the AYA cancer experience through actions and behaviors themselves. How these suggestions and information are disseminated may differ based on a number of factors including, but not limited to, clinic flow, provider roles, staffing, and availability. A few potential avenues to disseminate this data could be through offering AYA-specific trainings, utilizing AYA leadership panels, or hosting AYA focus groups.

The findings can also be used to provide information and suggestions to AYA cancer patients, as well as their friends and family members. AYAs frequently describe feeling isolated and ostracized during their cancer experience [7,10,40]; therefore, having this data can help normalize and validate their experiences and remind them that other patients share similar experiences. Providing this information for AYA patients also allows them to concretely identify ways that others can help and reinforces how they may already be helping. These suggestions can allow AYAs to elicit additional support and communication from various sources, resulting in increased self-efficacy. Similarly, parents or friends of AYA patients may be unsure about how to best help during treatment. The findings can inform and facilitate discussions about how family and friends can be helpful, or reinforce behaviors that are already helpful. Future directions about how this information can be operationalized will be discussed in sections below.

Our study also highlights how AYA patients can differ in their preferences throughout treatment, as indicated by the somewhat contradictory themes. For example, AYAs appear to want autonomy and independence, but appreciate help with daily tasks from their parents. They express the desire for privacy, but also value physical presence and communication. These findings underscore the importance of maintaining open communication with the AYA patients about their preferences and needs throughout the course of treatment and asking them about both individual and social preferences, which may change frequently.

#### 4.2.2. Implications for Screening

The study findings indicate key points of interest relevant to conducting psychosocial screenings with newly diagnosed AYA cancer patients. The need for cancer centers to implement screening in order to identify patients and families who may be at greater risk for negative psychosocial outcomes has been consistently supported in the literature [41,42]. Still, few validated approaches exist as medical centers use both formal (e.g., questionnaires, measures) and informal (e.g., semi-structured clinical interviews) methods for screening [41,42]. Research suggests that screeners should include questions based on key research findings [41]. The findings of our study indicate that screening for quality of social support (e.g., “whom do you receive support from?” “how do they provide support?” “how frequently do you communicate/see one another?”) and level of communication (e.g., “do you tell your mother/friend what you need?” “have you spoken to your social support about what is helpful?”) are both important factors when considering AYA psychosocial risk, yet few screeners appear to address this need. Even screens that are comprehensive and validated in pediatric settings, such as the Psychosocial Assessment Tool (PAT) [41,42], do not appear to adequately assess for characteristics of social support.

Further research is needed to examine psychosocial screening with AYA cancer patients, but ought to include detailed questions regarding the presence and actions of social supports to identify those patients who may be at risk for worse psychosocial outcomes. Those patients who appear to have minimal or less active social supports could be identified and targeted for intervention (e.g., Psychology, Child Life, Social Work, Music Therapy, family therapy, organizational support) or education. As such, medical staff may have a unique opportunity to foster coping in psychosocially at-risk AYA cancer patients by connecting them to appropriate resources and/or interventions.

#### 4.2.3. Implications for Intervention

The results of this study also provide implications for intervention tailored to increase social support through cancer peers, friends, or family members. The positive impact of peer (e.g., same-aged cancer patient) support has been documented in the literature [34,36,40] and findings from this study are consistent with these claims about cancer peer support, yet programs struggle to build and maintain support groups for AYA cancer patients. Many barriers seem to exist when trying to develop such programs [38,40], but the highlighted importance of peer support is unmatched. More research is needed to address the implementation and retention of AYA peer support programs, and oncology centers ought to consult with patients and families to identify ways in which support groups, peer mentorship, and leadership programs can be developed and maintained. Meeting other cancer patients was described as both one of the most helpful social interactions during treatment as well as the most desired experience for AYA cancer patients. Therefore, peer implementation should be a priority for the psychosocial care of AYA cancer patients. Organizations or camps that facilitate meet-ups and social events for younger pediatric patients may expand their age cutoff to include AYAs. As AYA cancer patients strive for “normalcy,” AYA events should include developmentally appropriate activities and topics. Given that communication was described as a helpful form of social support, connecting with peers via social media, online forums, or phone applications might be a promising pathway to fostering peer support. To address AYA patients’ appreciation of continued friend support, planned events/camps/meet-ups should allow patients to bring friends or family members to promote growth in all social relationships.

Families or AYA patients who struggle with communicating their needs with others may benefit from individual, family, or group psychotherapy to address these needs. Specifically, intervention topics may address family or interpersonal dynamics, including the categories and themes found in this study (e.g., presence, independence, support with daily tasks, positive distraction, maintaining a positive attitude, communicating with others, etc.).

### 4.3. Future Directions

Based on findings from this study, future directions include research related to the clinical implications discussed in above sections: disseminating information, addressing gaps in screening procedures, and implementing interventions targeted at improving social support. One way to provide this information to AYA patients could be through feasible and low-cost avenues such as informational brochures or worksheets. Research has indicated that using handouts with verbal reinforcement has been effective in teaching parents about adolescent behavior [43], increasing compliance with medication-taking [44], and that handouts create a more effective learning environment [45]. Using the data collected in this study, authors have developed an interactive AYA Coping Worksheet, for use between a provider and patient. This AYA Coping Worksheet has been presented to an AYA Advisory Board for evaluation and feedback from AYA patients. It is currently being piloted with AYA cancer patients for additional feedback and modification. The purpose of this worksheet is to validate and normalize the AYA experience, provide suggestions for positive coping based on other AYAs’ experiences, and to increase AYA patient self-efficacy to implement self-coping strategies or improve communication to increase social support. As worksheets are a feasible and accessible tool, they are intended to be used more universally with AYA patients, not just those identified as being psychosocially at risk. Future studies may examine the impact of this information on AYA patient coping and psychosocial outcomes.

The piloted worksheet was developed for use with AYA cancer patients, but additional worksheets are being developed to use with family, friends, or other sources of social support. Future directions also include creating an informational brochure for medical team, summarizing the findings of this study to increase their awareness of AYA developmental needs and intervention. Future studies could also examine the impact this information or training has on medical provider’s knowledge of AYA concerns.

Future studies could also examine the impact of incorporating social support into AYA psychosocial screening. Perhaps these studies could examine the relationship between level of social support and psychosocial outcomes, and determine if AYA patients with limited social support are at higher risk for negative sequelae. Future studies may also implement developmentally adapted interventions that incorporate social support, either in addition to pre-existing evidence-based interventions or as an independent form of intervention. Future interventions to be examined may include activities to improve communication skills within the family, family therapy, group therapy, or family/group educational sessions depending on the needs of the AYA patient. Similarly, conducting intervention or educational sessions with AYA patients’ friends, spouses, or classmates/colleagues may be a novel approach to improving AYA functioning. Future research might examine the role of peer support groups or mentorship programs in this age group, and address barriers and challenges with implementing these groups. Lastly, research may be conducted to examine how institutional or administrative changes may improve social support. These changes might include extending visitation hours, clustering care to allow for AYAs to have increased privacy either by themselves or with their friends, having AYA designated clinic days or recreational spaces, or offering social functions/events specifically for AYAs.

Future research is also needed to address underrepresented groups in this study as described in the limitations below, such as conducting interviews with non-English speakers and a greater number of female patients. Research including a broader array of subjects is recommended. Additionally, future research ought to examine the role of spouses as caregivers to understand how or if their perspective and experience differs from other forms of AYA social support during treatment. A small percentage of screened eligible participants (*n* = 4) had spouses, and the one spouse that was approached, declined interest in participating in the study. The findings from this study regarding spouses/significant others, although limited, suggest that they have a unique role in assisting AYAs coping with cancer, which may differ qualitatively from friendships and peer social support. Data from the two participants who described spousal support indicate the quality of this relationship is deeper and meaningful; however, not enough information was collected regarding this social group to relay suggestions or determine themes. Conducting research with AYAs and their spouses would be a novel study and supplement the social support literature.

Similarly, future research ought to examine the treatment and diagnostic factors of AYA cancer patients in order to examine potential differences in experiences and coping. Participants in this study presented with a relatively wide range of diagnoses, prognoses, and time since diagnosis, with two participants being treated for relapse or secondary cancer. Although responses in this study did not appear to differ based on these medical factors, the sample size was small and future studies would benefit from examining experiences in a broader array of patients, specifically factors such as diagnosis, length of treatment, relapse, treatment intensity, and prognosis.

Lastly, this study should be replicated with AYAs in other oncology settings, including with AYAs treated at adult oncology hospitals or clinics in order to provide a greater understanding of the AYA cancer experience. Research has found that AYA cancer patients treated in adult oncology clinics have worse outcomes (e.g., higher risk of relapse and fewer months of maintenance therapy) than those treated in pediatric clinics [46], so interviews with AYAs in both settings would increase generalizability and provide greater understanding of how this age group copes in a variety of settings.

### 4.4. Limitations

One of the main limitations of qualitative approaches is that the findings cannot be extended to wider populations with the same degree of certainty as appropriately powered quantitative analyses [47]. Although there was some within-group variability in medical and diagnostic factors (e.g., cancer diagnoses, prognoses, time since diagnosis, and relapse/recurrence rates), the sample was relatively homogenous demographically and in psychosocial functioning; the AYA group was predominantly male (70%) with private insurance (70%), while the parent group was predominantly female (80%) with public insurance (70%). As part of the inclusion/exclusion criteria, all participants were required to be proficient in speaking English. Although groups were combined for overall data analysis and no significant differences were found between the AYA group and the AYA children of the caregiver group, they might have represented different ethnic, cultural, and socioeconomic backgrounds. More female AYAs declined participation in the study after being approached (*n* = 5) versus males AYAs who were approached (*n* = 2), perhaps indicating that AYA female patients cope differently with treatment than AYA male patients or prefer not to share their experiences during treatment. Regarding cultural limitations, the adolescent and young adult developmental trajectories described in this study are also largely influenced by Western research and ideas about individuation, separation from parents, independence, and privacy; therefore, the results may not be applied to other cultural groups where this process may differ based on familial expectations. In fact, a cross-cultural psychology study found the experience during adolescence is variable and contingent upon culture, as some cultures have lengthier adolescent stages, while some do not describe an adolescent developmental stage at all [48]. Therefore, the findings regarding this age cohort and the desire for “normative” AYA development may only be applicable to cultures who share similar beliefs and ideas about adolescence and young adulthood.

As with most research, the subjects were a self-selecting group who chose to participate in the study. Participants scored in the high to average range on all measures. As such, results were analyzed with the assumption that these are experiences of AYAs who exhibit high hope and who felt “well enough” to participate in the study. Less may be generalized to those who were experiencing extreme treatment complications, on palliative treatment, or who did not wish to participate in the study.

Due to time constraints and availability, the primary researcher conducted 90% of the interviews. While this lends consistency across our data collection, we may have sacrificed variation in style and rapport that could have enriched the depth of interview data. Although coding themes and processes were discussed in weekly meetings and other measures were implemented to reduce researcher biases, the raw data was viewed independently by the primary researcher such that the investigator’s clinical and research background may have influenced the development of the coding framework. However, we attempted to mitigate this influence through study team discussion of both coding and interpretation.

## 5. Conclusions

It is apparent that social support is a key component of the coping process for AYAs undergoing cancer treatment. Different sources of social support address different challenges and stressors described by AYAs. Although cancer treatment clearly disrupts the trajectory of AYA development and individuation, social support appears to be a route in which AYAs can maintain some form of normalcy and growth towards independence, individuation, strong peer relationships, and identity exploration. All sources of social support ought to be informed about these developmental needs in order to partake in helpful and appropriate behaviors that assist with AYA cancer patient coping. In order for social support to be effectively operationalized, all participants would benefit from increased understanding, knowledge, and education about helpful, supportive activities. Improving the ability, awareness, and understanding of AYA patients’ social supports has the potential to enhance AYA patient coping and improve their psychosocial experience during cancer treatment for this at-risk and understudied age cohort.

## Figures and Tables

**Table 1:** Participant demographics.

Demographic Variable	Total Sample	AYA	Caregiver	AYA of Caregiver
	*n* (%)	*n* (%)	*n* (%)	*n*
Age				
Mean (*M*)	32.2 years	18.9 years	45.6 years	16.2 years
Standard Deviation (*SD*)	13.9 years	3 years	4.9 years	3.9 years
15–17	4 (20%)	4 (40%)	-	7
18–23	4 (20%)	4 (40%)	-	3
24–29	2 (10%)	2 (20%)	-	-
30–35	-	-	-	-
36–41	2 (10%)	-	2 (20%)	-
42–47	4 (20%)	-	4 (40%)	-
48 and older	4 (20%)	-	4 (40%)	-
Sex				
Male	9 (45%)	7 (70%)	2 (20%)	6
Female	11 (55%)	3 (30%)	8 (80%)	4
Race				
White	11(55%)	4 (40%)	7 (70%)	7
Black	7 (35%)	4 (40%)	3 (30%)	3
Asian	1 (5%)	1 (10%)	-	-
Other	1 (5%)	1 (10%)	-	-
Ethnicity				
Hispanic	5 (25%)	2 (20%)	3 (30%)	3
Non-Hispanic	15 (75%)	8 (80%)	7 (70%)	7
Insurance ^1^				
Public	10 (50%)	3 (30%)	7 (70%)	7
Private	10 (50%)	7 (70%)	3 (30%)	3
Education				
Partial high school	6 (30%)	6 (60%)	-	8
High school graduate	4 (20%)	1 (10%)	3 (30%)	2
Partial college	3 (15%)	2 (20%)	1 (10%)	-
Standard college graduate	4 (20%)	1 (10%)	3 (30%)	-
Graduate professional training	2 (10%)	-	2 (20%)	-
Marital Status				
Married	6 (30%)	1 (10%)	5 (50%)	-
Single	11 (55%)	8 (80%)	3 (30%)	10
Separated	1 (5%)	-	1 (10%)	-
Divorced	1 (5%)	-	1 (10%)	-
Other	1 (5%)	1 (10%)	-	-
Employment Status				
Working full-time	5 (25%)	1 (10%)	4 (40%)	-
Working part-time	2 (10%)	1 (10%)	1 (10%)	1
Homemaker	3 (15%)	-	3 (30%)	-
Unemployed	1 (5%)	1 (10%)	-	2
Temporarily laid off (FMLA)	3 (15%)	2 (20%)	1 (10%)	-
Student	5 (25%)	5 (50%)	-	6
Failed to respond	1 (5%)	-	1 (10%)	-
Disabled	-	-	-	1

^1^ Insurance was gathered from patient EMRs, and therefore describes the AYA’s insurance status.

**Table 2:** Results from descriptive measures.

Measure	AYA		Caregiver	
	*M*	Interpretation	*M*	Interpretation
AHS (raw) ^1^	range = 8–64			
Total Hope	58	high	56.3	high
Agency	27.2	-	27.2	-
Pathways	28.6	-	28.6	-
DASS-21 (raw) ^2^	range = 0–7			
Depression	3.8	normal	-	-
Anxiety	3.9	mild	-	-
Stress	6.9	normal	-	-
PedsQL-C (SS) ^3^	range = 0–100			
Total	72.6	average	68.7	average
Physical	59	average	64	average
Emotional	79.6	average	72.8	average
Cognitive	73.3	average	71.4	average
Social	74	average	72.9	average
MPSS (raw) ^4^	range = 1–7			
Total	5.7	high	-	-
Family	5.9	high	-	-
Friends	5.5	high	-	-
Significant other	5.6	high	-	-

^1^ The Adult Hope Scale. ^2^ The Depression Anxiety Stress Scale-21. ^3^ The Pediatric Quality of Life Inventory 3.0—Cancer Module, (Scaled Score). ^4^ Multidimensional Scale of Perceived Social Support.

**Table 3:** Family support.

General Support	Q1: “I think family is probably the most important thing because, you know, friends come and go, but family are always there for you. And that, I think you really, you pick up on that when you’re going through this and, um, so I think making sure you have family that are, that are close to you and that can be there for you and, um, you know provide emotional support, um, is a really big deal with getting, with getting through this” (2A) ^1^
	Q2: “Just being here for me and making sure that I’m okay mentally and emotionally to the best of their abilities. They really go above and beyond for me to the best they can” (8A)
Presence	Q3: “It really helps knowing that I have her there and mostly all the time so, I know a lot of kids my age, you know, don’t have that. And she also stays in the hospital with me when I’m here and she spends the night, so you know having someone there all the time I think really helps because you do get lonely in the hospital and even when you’re at, when you’re at home, ‘cause I’m not able to go to college or have a job right now so having someone to talk to you about a certain things really helps” (2A)
	Q4: “She really likes it when we’re here. Just to be here is important for her” (13C)
Everyday Tasks	Q5: “And I’m very happy to do things for him. And I think maybe I shouldn’t do that as much. To let him, you know, do things that he can. I know that there are some things that he just can’t do. A lot of times he’ll ask me to open a jar or something for him because he just, he can’t grip it” (13C)
	Q6: “She helps me get up and use the restroom. I’m kind of wobbly, so she helps me with that” (6A)
	Q7: “Just to kind of help her with the day-to-day things like, you know, if she’s especially when she’s in pain, she likes someone to be there. Um, or to help her with food like ordering the food in the hospital. Um, go to the bathroom. Kind of get changed. Um, Yeah. Just things like that” (12C)
Maintaining Autonomy	Q8: “He needed his space, we needed our space. Like now I’ll say, ‘I’m going to go get my breakfast.’ And he’ll be like, ‘Yeah you don’t have to hurry back.’ I’m like, ‘Okay.’ […] I’m trying to protect his privacy, um, that’s the other thing is protecting his privacy. So we kind of tried to establish our boundaries to start with” (16C)
	Q9: “That made a huge difference. Having those couple of hours of privacy, for him to connect with his friends and talk silly […] so now, I’m taking the liberty to leave the room. I’m taking the liberty to walk around, uh, the hospital and give him, uh, some, uh, space. And he’ll call me if I’m gone too long, he’ll call me” (20C)
Advocate	Q10: “It’s nice that she’s here so that when the doctors come in and she can listen and ask questions and make sure a double, like we said earlier, a double set of ears” (5A)
	Q11: “I also contacted the, um, uh, the psychologist here and she came in and talked to him and, um, and I said, ‘use all the tools that are available to you if, you know, if you, if you think she will be better, use her. Um, if you just need somebody to pray for you, you know who to call, and I just want you to have every tool’” (16C)
	Q12: “I’m the one who has to say, ‘Oh, well last week when he had the lumbar puncture, he had a few rough days after that.’ And he’s like, ‘Oh yeah I forgot’” (17C)
Positive Attitude	Q13: “‘Cause my dad, gosh, he’s like the silliest person ever. But I feel like he’s the reason why I can keep going because even if things should be sad, he doesn’t make it sad. He really doesn’t make it sad, he’s just, he can never stop joking” (8A)
	Q14: “So then I had to pick up the slack and I had to encourage him and I had to let him know, ‘hey, you know, we’re gonna, uh, face this. And we’re gonna be good.’ And, uh, ‘so whatever you read on the internet, you know, that’s, that’s their report. But we have a better report. And this is what’s going on, and this is what we’re going to do’” (20C)

^1^ “A” = AYA participant; “C” = Caregiver participant; corresponding numbers represent the unique participant codes.

**Table 4:** Friend support.

Category	Quotes
Presence	Q15: “I’m kind of limited on what I can do, I can’t like go out and hang out with all my friends like I used to, you know, but when they come and see me I think, I think that’s really helpful ‘cause, you know, it’s gives me time to interact with my friends and someone my age” (2A)
	Q16: “Sometimes they bring me stuff like food, because they know I get hungry sometimes, you know. And … I don’t know, just keeping me company in general” (8A)
Communication/Contact	Q17: “At the beginning they just messaged me stuff. Just saying how I’m doing and stuff […] It’s kind of like knowing that [they’re] there feels good in a way, yeah. Seeing that you’re not exactly alone in this process” (1A)
	Q18: “I do I think they helped. Even though they weren’t always here physically or anything, like it, luckily he had his cell phone […] he could text them different things” (11C)
Distraction	Q19: “There was one time, um, this friend of his came over, a young lady. And she brought food and movies to watch. And I mean, he was having a great time. It was really good” (17C)
	Q20: “They bought sushi […] Um, and so they just came by, ate, watched movies and stuff like that” (18C)
Encouragement/Gestures	Q21: “his friends gave him cards and stuff […] made him realize that he still was important to them I think” (11C)
	Q22: “His Young Life buddies, there were like 60 of them that all got together and FaceTimed and said, ‘Oh we missed you’ […] and ‘we’re praying for you’ […] and they all signed a big poster board and he was pretty excited about that” (16C)

**Table 5:** Other social support.

Source	Quotes
Peers	Q23: “I met some really nice people that have had, have had the same cancer as I have right now, which is not very many, but meeting people that have been through it and them giving you tips, you know, on how to get through, um, when you get delayed for chemo and, um, get off schedule, and um, you know, when you, when you get, had to go to the ER for a fever at 2 in the morning, you know, things like that. You know, like how did you make it through that without losing, you know, all your frustration or whatever and so they gave me, I guess they gave me more hope that I, that I can make it through this so, uh, they’ve been, they’ve been really helpful” (2A)
	Q24: “With the little game room area, the teenage game room area, I know he, he met some kids and talked to them and went to a couple those little meetings with them and what have you, and I think that probably helped a lot to know how other kids were dealing with it and how they were going through it” (11C)
	Q25: “I want to talk to somebody my age because uh, uh, it’s, so, so I can kind of relate to, to, I can kind of relate instead of like talking to like a younger kid” (3A)
	Q26: “I mean it would, it would […] not only it would show her of course she’s not the only one, and people are facing the same thing, the same situation […] it seems like they bond better. Yeah because there’s an understanding, shared experience” (19C)
Medical Staff	Q27: “The medical team seem to know what they’re doing and I trusted them to get it done correctly” (5A)
	Q28: “So uh [my doctor] was, you know, real with me from the beginning, you know, with not everyone survives cancer and so, you know, you have to know that there’s a chance that, you know, you may die from it or whatever and stuff, um, that, that, that was helpful” (2A)
	Q29: “One of the nurses brought in a backpack with a blanket and sleep pants and other comfort things. And it was wonderful” (16C)
	Q30: “And the, just the wonderful staff that’s here at Children’s also working with him and us I think helped them get through it” (14C)
Spouse/ Significant Other	Q31: “My fiancé’s been a big part of it. He helps me. He knows a lot more than anybody else, than anybody else, and he doesn’t let me just … He doesn’t go by with me just saying, ‘Oh yeah, I’m just having a bad day.’ He wants to know what’s going on […] and, and I actually tell because he can, he can brave through it. And he’s a very strong person, and he’s helped me so much. And sometimes whenever I just need somebody to give me a hug, help me through the day” (9A)
	Q32: “I mean she’s had a boyfriend for some time now and you know he’s been around, and she’s actually really close with his family and his mother. Um, I know I know when she’s not, when she’s feeling really bad and not feeling … She’s feeling bad, she likes to go over to his house and will stay the nights there” (12C)
School/Work	Q33: “You know some of [her] colleagues or her work, people from work have, um, put together like fundraisers for donations and stuff like that which has been helpful” (12C)
	Q34: “His teachers um they well his school in general is like they all you know rooting for him, rallying for him, um, his, the senior class um, uh, his teachers. […] Everybody is really trying to be very accommodating to make sure he graduates with the with the school you know” (13C)
Professional or Collegiate Athletes	Q35: “So we’ve had a lot of, like, professional athletes reach out to me and stuff. And I, like actually last week [a professional football player] came to my house and it was really cool to talk to him and I’ve gotten signed autographs from so many, so many players that I can’t even name. But, uh, yeah the sports, the sports players and teams have been really kind to me and that they’ve all kind of reached out to me and, you know, given me hope and, you know, knowing, telling me that, you know, they’re here for me and praying for me so it’s really nice to know that you have those kind of people supporting you as well as your family”(2A)
	Q36: “There so many good things that happen, I forgot like a [college football] team came up here one time and visited with him the [professional football team] came by one time. Uh, some of the [professional hockey] folks came through what have you […] College football players that came through […] and things of that nature that was just, uh, that was a, that was that was a wonderful thing for him” (11C)
Organizations	Q37: “But, um, we, we went with [a national organization] helped us a lot too we would go to games and stuff that he’d never been, or I’d never been, I had never been to the [professional basketball] game we went. That stuff helped him a lot. […] Especially when he was, like he was homeschooled so he had, for at least a year, he didn’t get to go anywhere, you know, anywhere unless [the national organization] invited him once a week, twice a week somewhere” (14C)
	Q38: “Then we met a wonderful group that was up here that was really helpful that was [a hunting organization], and he went and got to shoot around with them” (11C)
Community	Q39: “They, they send me Bible verses and they tell me to be strong and they remind me of times that I’ve had difficulty in the past and I’ve already gotten through it” (5A)
	Q40: “We met, I mean we met people on Facebook that are, you know, um, that that just want to do things for us like bring food over or um they send us like a shirt or you know just little things and it really means a lot when they do that” (2A)

**Table 6:** Opinions/advice.

Category	Quotes
Not Helpful	Q41: “I mean she doesn’t wanna do anything that reminds her of what’s happening. I mean we ended up still going, and someone like tried to talk to her or give her a shirt or something and she just really didn’t like that. She doesn’t like the fuss and she hates like people kind of giving her all that attention. You know doing that kind of stuff. So. Yeah that’s really not helpful and even in here I think she really just doesn’t want to hear about anything with her treatment or disease” (12C)
	Q42: “It’s kind of hard watching [my parents] argue over [my treatment], you know, ‘cause I don’t really need that right now” (2A)
	Q43: “Like nobody knew how to handle it. So every time they would talk to me, all they would talk about was being sick. It’s like, man. Like I’m still the same guy. We played, I’m still the same guy, we played football together since we was like seven, eight years old. I’m still, you know? I still like sports. I still like talk about other things. You know? We talked about, we, we, we didn’t, we didn’t become friends over being sick. So like how come every time we talk it got to be about that?” (4A)
	Q44: “And like, even my mom, first chemo she left me by myself. And she didn’t want my dad around ‘cause she doesn’t like seeing him, so she made my dad leave so she can come visit me. So he went to work ‘cause he thought she was gonna spend the day with me. As soon as they brought my first chemo out, I told them, ‘hey, can I wait for my dad?’ ‘Cause I want my dad to be here. And they said, ‘okay, sure.’ And my dad wasn’t showing up, it turned out the 18-wheeler broke down, ‘cause he drives an 18-wheeler. Um, he was waiting for somebody to come get him so he can come see me. And then so they said, ‘hey, we have to start soon.’ So they started. As soon as they start plugging me in, my mom just leaves”.(8A)
Advice to Others	Q45: “My advice would have to be like, would, would be try to treat that person as normal as possible. You know? There’s a lot more to me than just having cancer. You know? And that’s one thing that I will say. Like, don’t make, don’t make the person feel like they got cancer patient attached to their name” (4A)
	Q46: “it would be nice to see more resources for his age group. I think they kind of get lost. […] Like I saw a Facebook post for, by [the pediatric clinic]. I, you know, they have a page or group or whatever. And they posted something about the […] organization, and it had all these cool events that looked like he would even enjoy. There was like a [golf] thing, and a gaming thing. And a couple other events. I don’t remember what the other ones were. But they top out at 18…And I’m like, oh, he’s 20. You know? He would still enjoy doing stuff like this” (17C)
	Q47: “I wish that [my friends] knew how to help me more […] I wish they knew kind of like how to, how to relate to me more and understand what I’m going through […] I think I guess just being around me more and, um, you know, maybe coming to the hospital, and then seeing all this. I think when you go to the hospital and, you know, you, you see someone, you know, kind of going through this, um, it’s easier, you know, you understand it more, you know? […] and so, um, I guess if they came and saw me in the hospital they, they would probably you know, be like, ‘wow this, that’s, that’s something else’ having to, having to stay you know here all the time and go here, you know, all the time for blood work and stuff” (2A)
	Q48: “Um, I think that, um, I don’t know, I would say maybe in the future, um, I mean I know it’s, it’s limited or, you know, the hospital, but I would say like more so putting teens together and then putting little kids together just so maybe she could have talked to somebody else going through the same thing […] or having a you know teen day or something” (15C)
	Q49: “Just to, if the person wants to talk about it, like for anybody in general, if the patient wants to talk about it with you then let ‘em. Let ‘em go all out. Don’t really question ‘em while they’re going. If they want you to ask questions, you know, if they pause and they’re looking for the questions then ask as many as you want, but it’s good to just have somebody to listen to. So just sit there and listen and if they don’t wanna talk about it, then don’t pressure ‘em into it because that’s one of the worst things, and it’d be nice to just have a regular conversation and not be seen as somebody that’s, you know, don’t see just the cancer. See me as well” (9A)
	Q50: “When things come up, you need to talk to the patient and actually take, especially if they got, you know, take their feelings and their opinions and everything into consideration about what’s going on with them. Like, don’t have somebody speaking for them, for, you know? ‘What’s going on.’ ‘Hey.’ You know. ‘Okay, let’s talk to him.’ ‘Let’s see how he feels about this.’ You know? […] Basically, let’s not make them feel like, you know, they’re just so, let’s leave them, not make them feel helpless, you know?” (4A)

## Appendix A

### A.1. Interview Guide—AYA Form

Orientation statement: Our goal with this study is to understand how adolescents and young adults cope during cancer treatment. Our hope is that by talking directly to parents and patients, we can gain a better understand and in turn help those who are struggling to cope during treatment. There are no right or wrong answers—you are the expert in your life and in your experience.

1. Tell me how things have gone for you since you were diagnosed with cancer.
  - Has anything happened during this time that has changed things for you? If so, what?
  - What kind of things are you doing/have you done to get through this time?
  - How did you do that?
  - What has been a challenge so far and how did you handle it?
  - What have others done?
  - Why was it unhelpful/helpful?
  - What do you wish they would/would not do?
  - (Follow up: How do you think social support is related to your coping?)
2. However you think of hope, how is hope related to all of this?
  - Tell me about times you did not feel hopeful or you had low hope.
  - What do you hope for now?
  - How do you plan to get there?
3. What else should I know about your experience with cancer that I haven’t asked?
  - What is important for us to know?

### A.2. Interview Guide—Parent Form

Orientation statement: “Our goal with this study is to understand how AYA’s cope or deal with cancer. Our hope is that by talking to parents and patients, we can understand more and in turn help those who are struggling to cope during treatment. There are no right or wrong answers—you are the expert in your life and in your experience.”

1. Tell me how things have gone for your child since their diagnosis.
  - What kind of things is/has your child done to get through this time?
  - What has been a challenge for them so far and how did they handle it?
2. What have you done to help your child through this time?
  - What do you think your child wanted you to do/wanted from you?
  - How did these match or mismatch?
3. What have others done?
  - Why was it unhelpful/helpful?
4. However you think of hope, how is hope related to all of this?
  - What does your child hope for?
  - How do you think he or she will get there?
5. What else should I know about your child’s experience with cancer that I haven’t asked?
  - What is important for us to know?

## References

[1] Coccia P. (2019). Overview of Adolescent and Young Adult Oncology. J. Oncol. Pract..

[2] Schwartz L., Drotar D. (2005). Posttraumatic stress and related impairment in survivors of childhood cancer in early adulthood compared to healthy peers. J. Pediatr. Psychol..

[3] Barrera M., Shaw A.K., Speechley K.N., Maunsell E., Pogany L. (2005). Educational and social late effects of childhood cancer and related clinical, personal, and familial characteristics. Cancer.

[4] Rourke M.T., Hobbie W.L., Schwartz L., Kazak A.E. (2007). Posttrauamatic stress disorder (PTSD) in young adult survivors of childhood cancer. Pediatr. Blood Cancer.

[5] Kazak A.E., DeRosa B.W., Schwartz L.A., Hobbie W., Carlson C., Ittenbach R.F., Mao J.J., Ginsberg J.P. (2010). Psychological outcomes and health beliefs in adolescent and young adult survivors of childhood cancer and controls. J. Clin. Oncol..

[6] Felder-Puig R., Formann A.K., Mildner A., Bretschneider W., Bucher B., Windhager R., Zoubek A., Puig S., Topf R. (1998). Quality of life and psychosocial adjustment of young patients after treatment of bone cancer. Cancer.

[7] Abrams A.N., Hazen E.P., Penson R.T. (2007). Psychosocial issues in adolescents with cancer. Cancer Treat. Rev..

[8] Langeveld N., Stam H., Grootenhuis M., Last B. (2002). Quality of life in young adult survivors of childhood cancer. Support. Care Cancer.

[9] Kyngäs H., Mikkonen R., Nousiainen E.M., Rytilahti M., Seppänen P., Vaattovaara R., Jämsä T. (2001). Coping with the onset of cancer: Coping strategies and resources of young people with cancer. Eur. J. Cancer Care.

[10] Hokkanen H., Eriksson E., Ahonen O., Salantera S. (2004). Adolescents with cancer: Experience of life and how it could be made easier. Cancer Nurs..

[11] Ishibashi A. (2001). The needs of children and adolescents with cancer for information and social support. Cancer Nurs..

[12] Tillery R., Howard Sharp K.M., Okado Y., Long A., Phipps S. (2015). Profiles of resilience and growth in youth with cancer and healthy comparisons. J. Pediatr. Psychol..

[13] Woodgate R.L. (2006). The importance of being there: Perspectives of social support by adolescents with cancer. J. Pediatr. Oncol. Nurs..

[14] Hollingshead A.B. (2011). Four Factor Index of Social Status. Yale J. Sociol..

[15] Snyder C.R., Sympson S.C., Ybasco F.C., Borders T.F., Babyak M.A., Higgins R.L. (1996). Development and Validation of the State Hope Scale. J. Personal. Soc. Psychol..

[16] Snyder C.R., Harris C., Anderson J.R., Holleran S.A., Irving L.M., Sigmon S.T., Yoshinobu L., Gibb J., Langelle C., Harney P. (1991). The Will and the Ways: Development and Validation of an Individual-Differences Measure of Hope. J. Personal. Soc. Psychol..

[17] Lovibond S.H., Lovibond P.F. (1995). Manual for the Depression Anxiety Stress Scale.

[18] Osman A., Wong J.L., Bagge C.L., Freedenthal S., Gutierrez P.M., Lozano G. (2012). The depression anxiety stress Scales—21 (DASS-21): Further examination of dimensions, scale reliability, and correlates. J. Clin. Psychol..

[19] Szabó M. (2010). The short version of the Depression Anxiety Stress Scales (DASS-21): Factor structure in a young adolescent sample. J. Adolesc..

[20] Varni J.W., Burwinkle T.M., Katz E.R., Meeske K., Dickinson P. (2002). The PedsQL™ in pediatric cancer. Cancer.

[21] Ewing J.E., King M.T., Smith N.F. (2009). Validation of modified forms of the PedsQL generic core scales and cancer module scales for adolescents and young adults (AYA) with cancer or a blood disorder. Qual. Life Res..

[22] Zimet G.D., Dahlem N.W., Zimet S.G., Farley G.K. (1988). The multidimensional scale of perceived social support. J. Personal. Assess..

[23] Bradley E.H., Curry L.A., Devers K.J. (2007). Qualitative data analysis for health services research: Developing taxonomy, themes, and theory. Health Serv. Res..

[24] Glaser B.G., Strauss A.L. (1967). The Discovery of Grounded Theory: Strategies for Qualitative Research.

[25] Corbin J., Strauss A. (2008). Basics of Qualitative Research: Techniques and Procedures for Developing Grounded Theory.

[26] Glaser B.G. (1965). The constant comparative method of qualitative analysis. Soc. Probl..

[27] Morse J.M. (2015). Critical Analysis of Strategies for Determining Rigor in Qualitative Inquiry. Qual. Health Res..

[28] Decker C.L. (2007). Social support and adolescent cancer survivors: A review of the literature. Psycho Oncol..

[29] Gray R.E., Doan B.D., Shermer P., Fitzgerald A.V., Bery M.P., Jenkin D., Doherty M.A. (1992). Psychologic adaptation of survivors of childhood cancer. Cancer.

[30] Haluska H.B., Jessee P.O., Nagy M.C. (2002). Sources of social support: Adolescents with cancer. Oncol. Nurs. Forum.

[31] Banner L.M., Mackie E.J., Hill J.W. (1996). Family relationships in survivors of childhood cancer: Resource or restraint?. Patient Educ. Couns..

[32] Howard Sharp K.M., Willard V.W., Barnes S., Tillery R., Long A., Phipps S., Gerhardt C.A., Berg C.A., Wiebe D.J., Holmbeck J.N. (2016). Emotion socialization in the context of childhood cancer: Perceptions of parental support promotes posttraumatic growth. J. Pediatr. Psychol..

[33] Rolland J.S. (1987). Chronic illness and the life cycle: A conceptual framework. Fam. Process.

[34] Zebrack B., Chesler M.A., Kaplan S. (2010). To foster healing among adolescents and young adults with cancer: What helps? What hurts?. Support. Care Cancer.

[35] Robinson K.E., Gerhardt C.A., Vannatta K., Noll R.B. (2006). Parent and family factors associated with child adjustment to pediatric cancer. J. Pediatr. Psychol..

[36] Zebrack B., Bleyer A., Albritton K., Medearis S., Tang J. (2006). Assessing the health care needs of adolescent and young adult cancer patients and survivors. Cancer.

[37] D’agostino N.M., Penney A., Zebrack B. (2011). Providing developmentally appropriate psychosocial care to adolescent and young adult cancer survivors. Cancer.

[38] Treadgold C.L., Kuperberg A. (2010). Been there, done that, wrote the blog: The choices and challenges of supporting adolescents and young adults with cancer. J. Clin. Oncol..

[39] Parsons H.M., Harlan L.C., Schmidt S., Keegan T.H.M., Lynch C.F., Kent E.E., Xiao-Cheng W., Schwartz S.M., Chu R.L., Keel G. (2015). Who Treats Adolescents and Young Adults with Cancer/ A Report from the AYA HOPE Study. J. Adolesc. Young Adult Oncol..

[40] Cheung C.K., Zebrack B. (2017). What do adolescents and young adults want from cancer resources? Insights from a Delphi panel of AYA patients. Support Cancer Care.

[41] Kazak A.K., Brier M., Alderfer M.A., Reilly A., Fooks Parker S., Rogerwick S., Diltaranto S., Barakat L.P. (2013). Screening for Psychosocial Risk in Pediatric Cancer. Pediatr. Blood Cancer.

[42] Kazak A.E., Schneider S., Didonato S., Pai A.L. (2015). Family psychosocial risk screening guided by the pediatric psychosocial preventative health model (PPPHM) using the Psychosocial Assessment Tool (PAT). Acta Oncol..

[43] Hutchinson J.W., Stafford E.M. (2005). Changing Parental Opinions About Teen Privacy Through Education. Pediatrics.

[44] Robinson G.L., Gilbertson A.D., Litwack L. (1986). The effects of a psychiatric patient education to medication program on post-discharge compliance. Psychiatr. Q..

[45] Avval F.Z., Jarahi L., Ghazvini K., Youssefi M. (2013). Distribution of Handouts in Undergraduate Class to Create More Effective Educational Environment. Int. J. Educ. Res..

[46] Wolfson J.A., Sun C.L., Wyatt L.P., Hurria A., Bhatia S. (2015). Impact of care at comprehensive cancer centers on outcome: Results from a population-based study. Cancer.

[47] Atieno O.P. (2009). An analysis of the strengths and limitation of qualitative and quantitative research paradigms. Probl. Educ. 21st Century.

[48] Choudhury S. (2009). Culturing the adolescent brain: What can neuroscience learn from anthropology?. Soc. Cogn. Affect. Neurosci..

